# When Did Old Age Stop Being Depressing? Depression Trajectories of Older Americans and Britons 2002–2012

**DOI:** 10.1016/j.jagp.2017.06.006

**Published:** 2017-11

**Authors:** Gindo Tampubolon, Asri Maharani

**Affiliations:** Cathie Marsh Institute for Social Research, University of Manchester, Manchester, United Kingdom

**Keywords:** Depression, aging, cohort effect, English Longitudinal Study of Ageing, Health and Retirement Study

## Abstract

**Objective:**

This study aims to investigate the implications of the heterogeneous cohort composition on depression trajectories of older adults in the United States and England.

**Methods:**

Using growth curve models to identify depressive symptom trajectories and data spanning six waves over 10 years (2002–2012) from the U.S. Health Retirement Study and the English Longitudinal Study of Ageing, community-dwelling Americans and Britons aged 50 years and older were studied. Depressive symptoms were measured using the eight-item Center for Epidemiologic Studies Depression Scale.

**Results:**

The sample included 11,919 respondents (7,095 women [59.53%]) in the United States and 10,606 respondents (5,802 women [54.7%]) in England aged 50 and older. Older cohorts were shown to have higher depressive symptoms than younger cohorts in the United States and England. The trajectories of depression of older cohorts, particularly those of the prewar cohorts in both countries and the war cohort in England, followed a U-shape. Conversely, the trajectories of depression of the younger cohort, particularly those of the postwar cohorts in both countries and the war cohort in the United States, took an inverted U-shape.

**Conclusion:**

The trajectories of depression in later life between cohorts took different shapes. This finding may lead to the development of more cost-effective policies for treating depression in later life.

## Highlights

• We investigated the implications of heterogeneous cohort composition on depression trajectories of older adults in the United States and England, using the growth curve model to draw trajectories over a decade. The findings showed for the first time that the trajectories of depression in later life are radically different for different cohorts living at the same time in the early 21st century.
• The trajectories of depression of older cohorts, particularly those of the prewar cohorts in both countries and the war cohort in England, followed a U-shape. Conversely, the trajectories of depression of the younger cohort, particularly those of the postwar cohorts in both countries and the war cohort in the United States, took an inverted U-shape.

## Introduction

Population aging changes the composition of cohorts living in old age with implications that have largely been missed. Population aging is indicated by a larger share of people aged beyond the notional threshold of old age (Bismarck's 65 years) and by an expanding period of expectancy to enjoy this later life.^1^, ^2^ These two facts change the cohort composition of older people today. In the early 21st century, sizable numbers of prewar cohort members, war cohort members, and baby boomers intermingled. Members of each cohort can expect to live longer than their predecessors half a century before. These cohorts, with their compositions, sizes, and characteristics, can be expected to remain in society for some time because of their increasing life expectancy. Their composition matters for policy in a particular way and for our understanding of aging today.

Recently, reports have documented differential aging trajectories for different cohorts, particularly the trajectories of frailty,^3^ cognition,^4^ and well-being.^5^ Any uniform social policy on aging may therefore be appropriate for some cohorts but not for others. Basing policy on the experiences of the largest cohort or of a synthetic one, to the unintended neglect of others, may be inappropriate not only because of the size of the resulting mismatch but also because of the duration of its effect. After all, those neglected cohorts, with needs mismatched, are expected to live ever longer.

Depression is common among older adults,^6^, ^7^, ^8^, ^9^, ^10^ and its consequences are particularly severe. Later life depression may lead to higher suicide,^11^ disability,^12^ and morbidity and mortality rates^7^, ^8^, ^13^ among older people. Preventing depression is thus an important aspect of healthy aging to mitigate the challenges associated with globally aging populations.^14^ Cross-sectional studies have documented a significant nonlinear association between age and depression.^15^, ^16^, ^17^ Depression symptoms across life exhibit a U-shape: They are relatively widespread in early adulthood, decline during middle age, and rise again during old age. A more recent 10-year longitudinal study reported a consistent U-shaped association between age and depression among Canadians aged 65 and older after including risk factors.^18^ However, a longitudinal study using a community sample of U.S. respondents aged 65–95 found that the association between age and higher levels of depression diminished after taking into account the risk factors included in the analysis.^19^

In addition to documenting different trajectories of depression, the literature has also noted the well-being paradox of old age, according to which midlife adults' well-being is lower than in earlier and later life.^20^, ^21^ Although based mostly on recent cross-sectional data, this hints at the possibility that the postwar cohort may not follow their predecessors in experiencing a depressing old age. Suppose that both the prewar and war cohorts followed the synthetic trajectories of “old age is depressing,”^15^, ^16^, ^17^, ^18^ whereas the postwar cohort was more satisfied with their lot. If social policy were to consist of offering talking therapies to improve older people's mental well-being, then a synthetic trajectory-based policy would be insensitive and expensive.

Our purpose in this study was to investigate the implications of the heterogeneous cohort composition on depression trajectories from middle age (≥50 years) onward. We took advantage of the comparability built into the design of the U.S. Health and Retirement Study (HRS) and the English Longitudinal Study of Ageing (ELSA) to answer a series of research questions: Are different cohorts in America following different trajectories of depression? Are the same cohorts in Britain following comparable trajectories? What are the individual factors driving the different trajectories? Are there similarities and dissimilarities in those factors between the two countries?

This study showed for the first time that the trajectories of depression in later life are radically different for different cohorts living at the same time in the early 21st century: the U-shape intermingles with the inverted U-shape. It also revealed the period when one shape transitioned to the other, perhaps marking the unique role of the war in both the United States and England. Because the war cohorts followed different trajectories in the two countries, a new finding uncovered here, it offers rather different policy implications for maintaining the well-being and health of older people.

## Methods

### Data Sources

To identify within-person change in depression, we fit separate growth curve analysis to longitudinal data from respondents in the HRS (six biannual waves from 2002 to 2012)^22^ and ELSA (six biannual waves from 2002 to 2012).^23^ HRS data were harmonized with ELSA data; we chose those waves of the surveys because they had identical time frames and included the same measure of depression. The HRS data were collected using a combination of in-person and phone interviews, whereas all the ELSA interviews were performed in person. HRS collected information on the demographic, socioeconomic, and health circumstances of non-institutionalized adults aged 51 years and older in the United States, whereas ELSA collected the same information from non-institutionalized adults aged 50 years and older in England. To maximize the comparability of the U.S. and English sample, we included white respondents only because less than 5 per mil of the ELSA sample were nonwhites. Our final sample comprised 11,919 respondents in the United States and 10,606 respondents in England. Appendix 1 shows the frequency of samples based on age groups and cohort to show the sufficient overlap in the ages of the samples for each cohort in each data.

### Study Measures

#### Depressive Symptoms

The presence of depressive symptoms was assessed using the eight-item version of the Center of Epidemiologic Studies Depression Scale (CES-D).^24^ The CES-D has gained general acceptance as a tool to screen for depressive symptoms in older people^24^, ^25^, ^26^ and has been widely used in studies of late-life depression.^27^, ^28^, ^29^ Respondents responded to the eight questions regarding their feelings during the past week by answering “yes” or “no” to the following items: (1) you felt depressed, (2) you felt that everything you did was an effort, (3) your sleep was restless, (4) you were happy, (5) you felt lonely, (6) you enjoyed life, (7) you felt sad, and (8) you could not “get going.” A depressive symptom score was assigned by totaling all item scores after reversing the questions of the positive mood (Questions 4 and 6). The possible range of scores was 0–8.

#### Birth Cohorts

To ensure their comparability, we grouped the sample into three birth cohorts based on sociohistorical events having occurred in both the United States and England. The oldest cohort members, the prewar cohort, were born before the start of World War II in 1938, whereas the second cohort members, the war cohort, were born during the war. The most recent cohort members, the postwar cohort, were born in or after 1946. Although England came into the War in 1939 and the United States in 1941, we opted to ensure the same age range between the two data sets.

#### Covariates

A growing body of literature has investigated the relationships between demographic factors (e.g., gender, education, and socioeconomic status) and depression among older adults.^29^, ^30^, ^31^ The demographic variables used in this study were measured as follows. Age was determined by self-reported date of birth. Gender was included in the analysis with male as the reference. We categorized the levels of education completed by respondents into less than high school (reference), high school, and some college. Marital status was measured using a four-group categorization based on individuals' current and previous relationships: single, married or cohabiting, divorced, and widowed. We used tertiles of the aggregate of public pension wealth and private pension wealth to measure wealth,^4^ using the poorest tertile as the reference.

The literature has reported associations between behavioral risk factors and depression.^30^, ^32^ In this study we considered smoking status using the categories current smoker, former smoker, and nonsmoker (reference). In addition to smoking status, we included drinking behavior (drinking more than 5 days a week). Depression is known to be affected by physical and cognitive functions. Physical function in this study was measured by a summary score of (instrumental) activities of daily living, mobility, and muscle function. We assessed cognitive ability using tests of immediate and delayed recall of 10 words each. Because chronic conditions may be implicated in depression,^28^, ^30^ it is advisable to also include those conditions in the analysis. Chronic conditions included in this study were hypertension, heart condition, diabetes, stroke, arthritis, and cancer.

### Statistical Analysis

We evaluated trajectories of depressive symptoms over 12 years using a growth curve model, also known as random coefficients or mixed model. This model is a specific type of multilevel model in which the lowest level of observation is repeated over time using various measures that are then treated as nested within individuals.^33^, ^34^ This method is widely used in aging studies using longitudinal data.^4^, ^5^, ^19^ This model provides information on the effects of each determinant, including random and fixed effects, adjusting for all risk factors in the model. The growth curve analysis was carried out using four sequences of models. We began with a null model in which only age and squared age were included to identify the shapes or forms of the growth model. Next, we created a baseline model including age, squared age, gender, marital status, socioeconomic determinants, lifestyle factors (smoking status, drinking behavior, and physical exercise), and the presence of chronic diseases. Cohort indicators were included in the third cohort model. Finally, cohort indicators and the interaction between cohorts, age, and squared age were included in the fourth or final model. Akaike information criteria across the four models suggested the final model had the best fit. We then generated the predicted trajectories of depressive symptoms (controlled for all covariates in the final model) with separate curves for each cohort in the United States and England. All statistical analyses were conducted using Stata 14.0 (StataCorp, College Station, TX).

## Results

A total of 11,919 participants in the United States and 10,606 participants in England aged 50 years and over were initially included in this study. Table 1 shows the baseline characteristics of the American and English respondents in 2002–2003 and that older adults from the United States were, on average, less depressed than their English counterparts. There was a slightly higher proportion of male respondents in the English sample. The proportion of older adults who completed high school and/or attended college was higher in the United States than in England. A higher percentage of older adults in the United States suffered from every chronic condition included in this study. However, English adults reported less physical problems and more cognitive impairment. The percentage of English older adults who were smokers was a bit higher than those of American older adults, whereas the percentage of English older adults who drink regularly was slightly lower than that of American older adults.

**Table 1:** Baseline Characteristics of the HRS Wave 6 and ELSA Wave 1 Respondents, 2002–2003

	United States (N = 11,919)	England (N = 10,606)
CES-D score (SD)	1.37 (1.87)	1.57 (1.97)
Age, yr (SD)	68.23 (9.13)	64.81 (9.96)
Male	40.47%	45.3%
Cohort		
Prewar	66.78%	51.86%
War	26.81%	27.07%
Postwar	6.42%	21.07%
Marital status		
Single	2.31%	5.03%
Married	68.66%	69.7%
Divorced	8.99%	8.61%
Widowed	20.04%	16.66%
Education		
Less than high school	21.04%	57.84%
High school	35.2%	14.56%
College	43.76%	27.61%
Drink regularly		
Yes	90.87%	89.1%
No	9.13%	10.9%
Smoking status		
Never	41.1%	34.95%
Former	45.28%	46.94%
Current	13.62%	18.11%
Physical function (SD)	2.63 (3.17)	2.21 (3.21)
Cognitive function		
Low memory	62.79%	70.78%
Medium memory	25.74%	22.54%
High memory	11.47%	6.69%
Hypertension	47.73%	36.52%
Heart condition	23.35%	17.11%
Diabetes	13.80%	6.84%
Stroke	7.17%	3.95%
Arthritis	56.63%	31.5%
Cancer	13.98%	5.99%

*Notes:* SD: standard deviation.

**Figure 1:**
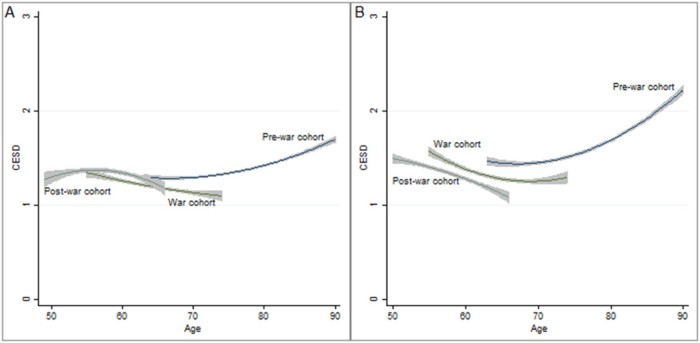
Predicted trajectories and 95% confidence intervals of depressive symptoms among older adults in (A) the United States and (B) England by cohorts.

Table 2 displays the results from the baseline and cohort growth curve models for depressive symptoms in the United States and England separately. The null model for depression includes curvilinear age effects only. It showed that trajectories in later life have curvilinear or quadratic shapes both in the United States and England. The curvilinear shape of the trajectories of depression in later life remained in the second and final models, where behavioral risk factors, chronic conditions, and birth cohorts were included. Cohort had different effects on respondents in the two countries. Model 3 presents the results for the association of birth cohort and depression among older adults. War cohort respondents were less depressed than prewar cohort respondents in both the United States and England. Respondents who were born after the war were less depressed than those who were born before the war in England, but the significance of that association did not appear in the United States. Interpreting the coefficients of age, squared age, cohort indicators, and interaction between them in the final model should be done simultaneously. Therefore, we predict the trajectories of each cohort and its interactions based on the final model (Figure 1) to better interpret the relationships between birth cohorts and depression.

(Source: HRS and ELSA 2002–2012.).

**Table 2:** Determinants of Depressive Symptoms Among Older Adults in the United States and England

	United States				England			
	Model 1	Model 2	Model 3	Model 4	Model 1	Model 2	Model 3	Model 4
Age	−0.133 (0.01)^a^	−0.047 (0.01)^a^	−0.046 (0.011)^a^	0.029 (0.023)	−0.192 (0.013)^a^	−0.097 (0.013)^a^	−0.108 (0.013)^a^	−0.07 (0.039)
Age^2^	0.102 (0.007)^a^	0.02 (0.007)^a^	0.017 (0.007)^b^	−0.03 (0.015)^b^	0.15 (0.009)^a^	0.057 (0.009)^a^	0.062 (0.009)^a^	0.037 (0.026)
Cohort, reference prewar								
War			−0.15 (0.029)^a^	4.855 (2.277)^b^			−0.109 (0.034)^a^	2.036 (2.871)
Postwar			−0.045 (0.039)	−3.017 (3.285)			−0.167 (0.042)^a^	−3.878 (3.189)
War × age				−0.131 (0.069)				−0.056 (0.086)
Postwar × age				0.128 (0.111)				0.142 (0.105)
War × age^2^				0.000 (0.000)				0.036 (0.065)
Postwar × age^2^				−0.001 (0.000)				−0.132 (0.089)
Female		0.158 (0.022)^a^	0.159 (0.022)^a^	0.159 (0.022)^a^		0.269 (0.025)^a^	0.27 (0.025)^a^	0.27 (0.025)^a^
Marital status, reference arried								
Single		0.308 (0.061)^a^	0.305 (0.061)^a^	0.304 (0.061)^a^		0.334 (0.053)^a^	0.335 (0.053)^a^	0.335 (0.053)^a^
Divorced		0.493 (0.03)^a^	0.495 (0.03)^a^	0.497 (0.03)^a^		0.44 (0.038)^a^	0.445 (0.038)^a^	0.446 (0.038)^a^
Widowed		0.551 (0.022)^a^	0.549 (0.022)^a^	0.549 (0.022)^a^		0.68 (0.03)^a^	0.676 (0.03)^a^	0.676 (0.03)^a^
Education, reference less than high school								
High school		−0.345 (0.029)^a^	−0.343 (0.029)^a^	−0.343 (0.029)^a^		−0.143 (0.035)^a^	−0.137 (0.035)^a^	−0.137 (0.035)^a^
College		−0.458 (0.029)^a^	−0.453 (0.029)^a^	−0.454 (0.029)^a^		−0.211 (0.029)^a^	−0.202 (0.029)^a^	−0.202 (0.029)^a^
Wealth tertiles, reference bottom								
Middle		−0.106 (0.017)^a^	−0.105 (0.017)^a^	−0.105 (0.017)^a^		−0.16 (0.021)^a^	−0.161 (0.021)^a^	−0.161 (0.021)^a^
Top		−0.147 (0.021)^a^	−0.146 (0.021)^a^	−0.146 (0.021)^a^		−0.282 (0.023)^a^	−0.285 (0.023)^a^	−0.284 (0.023)^a^
Physical problems		0.168 (0.002)^a^	0.168 (0.002)^a^	0.168 (0.002)^a^		0.169 (0.003)^a^	0.168 (0.003)^a^	0.168 (0.003)^a^
Cognitive function, reference low memory								
Medium memory		−0.082 (0.014)^a^	−0.082 (0.014)^a^	−0.081 (0.014)^a^		−0.136 (0.017)^a^	−0.13 (0.017)^a^	−0.13 (0.017)^a^
High memory		−0.106 (0.022)^a^	−0.105 (0.022)^a^	−0.105 (0.022)^a^		−0.14 (0.028)^a^	−0.129 (0.028)^a^	−0.129 (0.028)^a^
Drink regularly		0.023 (0.024)	0.022 (0.024)	0.022 (0.024)		−0.129 (0.028)^a^	−0.13 (0.028)^a^	−0.13 (0.028)^a^
Smoking status, reference never								
Former		0.088 (0.022)^a^	0.089 (0.022)^a^	0.088 (0.022)^a^		0.113 (0.025)^a^	0.115 (0.025)^a^	0.115 (0.025)^a^
Current		0.238 (0.03)^a^	0.242 (0.03)^a^	0.243 (0.03)^a^		0.304 (0.034)^a^	0.303 (0.034)^a^	0.303 (0.034)^a^
Hypertension		0.028 (0.017)	0.031 (0.017)	0.032 (0.017)		0.021 (0.021)	0.024 (0.021)	0.024 (0.021)
Heart condition		0.169 (0.019)^a^	0.169 (0.019)^a^	0.169 (0.019)^a^		0.161 (0.026)^a^	0.162 (0.026)^a^	0.162 (0.026)^a^
Diabetes		0.002 (0.022)	0.006 (0.022)	0.006 (0.022)		0.043 (0.035)	0.048 (0.035)	0.048 (0.035)
Stroke		0.071 (0.029)^b^	0.073 (0.029)^b^	0.073 (0.029)^b^		0.096 (0.048)^b^	0.097 (0.048)^b^	0.097 (0.048)^b^
Arthritis		0.068 (0.018)^a^	0.071 (0.018)^a^	0.072 (0.018)^a^		0.169 (0.022)^a^	0.175 (0.022)^a^	0.175 (0.022)^a^
Cancer		0.075 (0.022)^a^	0.076 (0.022)^a^	0.076 (0.022)^a^		0.132 (0.035)^a^	0.137 (0.035)^a^	0.137 (0.035)^a^
Constant	5.582 (0.385)^a^	3.171 (0.368)^a^	3.327 (0.41)^a^	0.273 (0.912)	7.584 (0.45)^a^	4.829 (0.444)^a^	5.429 (0.482)^a^	3.955 (1.5)^a^
Akaike information criteria	247071	238846	238822	238806	188490	165726	165714	165717

*Notes:* Values coefficients with standard errors in parentheses from the three growth curve models for each dataset. Significance of these coefficients was determined with a t statistic. Degrees of freedom ranged from 5 to 31 for both datasets.

a Significant at 1% or less.

b Significant at 5% or less.

The predicted trajectories of depressive symptoms (based on the final model) for the three cohorts are given in Figure 1. Those estimated trajectories represent the quadratic effects within birth cohort and country. These plots show for the first time the cohort effects in depression in the United States (Figure 1A) and England (Figure 1B). It shows that the prewar cohort had higher levels of depressive symptoms than the other cohorts in both the United States and England. In both countries the level of depressive symptoms of the prewar cohort increased as the respondents aged, following the U-shaped hypothesis. Conversely, the level of depressive symptoms of the postwar cohort slightly increased in the decade after reaching age 50 and then decreased after the respondents reached age 60 in the United States (Figure 1A) and decreased after the respondents reached age 50 in England (Figure 1B). A puzzling result is shown in the war cohort. In the United States the war cohort anticipated the depressive symptoms trajectories of the postwar cohort, whereas the war cohort in England followed the trajectories of the prewar cohort.

Several potential confounders and sociodemographic characteristics show similar significant associations with depression among older adults in the United States and England. In both countries women had higher levels of depressive symptoms than men. Single, divorced, and widowed respondents had higher depressive symptoms than married respondents. Higher educational attainment and better economic status were correlated with lower levels of depressive symptoms. Current and past smokers had more depressive symptoms than nonsmokers. Respondents with better cognitive function had fewer depressive symptoms. The analysis on the ELSA data showed that drinking alcohol more than 5 days per week was associated with higher levels of depressive symptoms. Conversely, the association between drinking alcohol more than 5 days a week and the level of depressive symptoms was not significant in the analysis on HRS data.

## Discussion

Using repeated assessment of the CES-D over 12 years, we modeled the trajectories of depressive symptoms among older adults in the United States and England. Three major findings emerge from our study, extending the literature. First, older cohorts had higher levels of depressive symptoms than younger cohorts after adjusting for education and risk factors. Second, the distinguishing mark of later life today is heterogeneity of its trajectories, reflecting its heterogeneous cohort composition. For the prewar cohort, depressive symptoms are associated with older age. This finding supports previous studies, which found that depressive symptoms follow a U-shaped trajectory.^15^, ^16^, ^17^ However, for most (the baby boomers), old age is not depressing, a finding that follows the inverted U-shaped trajectory of depression symptoms.^20^, ^21^ Nevertheless, old age has not entirely ceased to be depressing. The striking finding is that trajectories of depression in old age are now heterogeneous.

Finally, to answer the question of when old age ceased to be depressing, we need to focus on the war cohort in the two countries. In the United States the war cohort pattern presaged that of the baby boomers. The war cohort took a new path on which the baby boomers soon followed. In England the war cohort stayed on the old path that the baby boomers soon abandoned. The different trajectories of the war cohort in two different countries may be explained by different effects of war in those countries. Previous studies have shown the significant effect of World War II as a life event on depressive symptoms.^35^ It is instructive to compare children and adults from the Midwest America and the Midlands England. The theater of war in Europe involved adults from both the Midwest and the Midlands: Both were members of the prewar cohort, and both have displayed similar trajectories of depression. But the children were different. The children from the Midlands were more directly affected by the war, and the war children of the Midlands followed the shape of trajectories earlier traced by the prewar cohort. However, the war children of the Midwest reshaped the trajectories to be followed by the postwar cohort.

This contrast opens up a new way of understanding various trajectories of health and well-being in later life in these countries. The trajectories may be so heterogeneous that they move beyond what can be characterized as better or worse. The shapes are fundamentally different: U-shaped versus inverted U-shaped. Moreover, explaining this difference in terms of war experience, although not a complete explanation, may be a fruitful one. We hypothesize that trajectories of health and well-being in European countries can be arrayed along a continuum marked by the patterns observed in England at one end and by those in the United States at another. We hypothesize, for instance, that the experiences of German and French people will reveal late-life trajectories more similar to those of the English, whereas the Swedish war experience will render its trajectories of elderly well-being more akin to the American ones.

The strengths of our study include the wide range of potential covariates included in the analysis. Depression symptoms tend to be more severe among women, respondents who are not in a marriage or relationship, respondents with less education, and less wealthy respondents. These results provide additional support for the well-documented link between demographic factors and depression. A previous study using panel data from 11 European countries showed the significant relationship between physical activity and lower levels of depressive symptoms.^36^ Our finding that respondents with physical problems had higher levels of depressive symptoms in both countries supports previous research citing the fact that physical problems may restrict respondents' ability to perform physical activities.^37^ Despite known associations between depression and chronic diseases, we found in both countries that heart diseases, stroke, arthritis, and cancer were significantly associated with higher depression symptoms but that hypertension and diabetes were not. A study using data from 7,240 older women in four areas in the United States (Baltimore, Minneapolis, the Monongahela Valley, and Portland, Oregon) found that persistently high and increasing depressive symptoms were found in respondents with obesity, diabetes, and myocardial infarction.^30^ Patients with chronic diseases are likely to face numerous threats and challenges, including pain, disability, changes in lifestyle, and death. Facing such conditions may result in a variety of responses, including depression.

This study is subject to some limitations. First, the measure of depression used in this study (the CES-D) is a subjective measure, not a clinical diagnostic measure. However, the CES-D has been widely used and is well accepted for the modeling of depressive symptoms among older adults.^24^, ^27^, ^28^, ^29^ Second, our results may be affected by attrition. The literature has highlighted that the attrition in longitudinal studies in the field of aging is problematic, given that individuals who drop out of the study are more likely to be frail, less healthy, older, and poorer.^38^, ^39^ Because those dropouts cannot be assumed to be “missing at random,” subsequent studies should take attrition in the data into account to avoid bias in analysis. Third, there is only a limited overlap in age of assessments across the three cohorts because only the ages 61–65 are covered in all three birth cohorts. The differences across the cohorts in the shape of the depressive symptoms trajectories thus may be due to the constant variation across age. Given the ongoing nature of both HRS and ELSA, further study can rectify this limitation more firmly. Finally, this study pooled men and women together in the analysis. Literature has shown that men and women had different patterns of suicide rates.^40^, ^41^, ^42^ Further research can fruitfully explore the interaction between gender, cohort, and depression.

Our study has theoretical and practical implications. In the theoretical and empirical literature there is an unsettled debate regarding trajectories of health functioning in later life, whether they are largely marked by decline or essentially linear once comorbidities are acknowledged. The literature has begun to document improvement or deterioration across the cohort. No study, however, has reported that later life today brings improvements for one cohort and deterioration for another. Researchers will now be called on to recognize the radical and heterogeneous trajectories of later life; they are radical in that not only do the levels differ, but the directions flip from upward to downward within a society. Failure to appreciate this cohort effect is bound to lead to misleading inference.

Beyond the theoretical implications, this cohort effect has policy implications, leading to the consideration of more cost-effective policies. Treating depression requires considerable resources. For example, cognitive behavioral therapy, as one of the effective treatments for depression, is complex and depends on costly and highly trained professionals.^43^ For a sizable proportion of the older population (i.e., baby boomers), therapy may not be needed or can at least, perhaps, be postponed. Only a reduced number of older people need therapy. In short, more refined targeting (cohort versus period) may be both possible and easy to administer.

## Figures and Tables

**Table:** 

Age (yr)	HRS			ELSA		
	Prewar	War	Postwar	Prewar	War	Postwar
50–55	0	0	936	0	0	2,431
56–60	0	1,544	170	0	1,617	261
61–65	1,160	1,310	0	781	797	0
66–70	2,157	0	0	1,553	0	0
71–75	1,767	0	0	1,310	0	0
76–80	1,460	0	0	987	0	0
81–85	1,045	0	0	658	0	0
86–90	370	0	0	211	0	0
>90	0	0	00	0	0	00
